# Transcriptomic profiling and RANKL/RANK/OPG-mediated osteoclastogenesis in zebrafish larvae under simulated microgravity conditions

**DOI:** 10.3389/fcell.2026.1786373

**Published:** 2026-05-04

**Authors:** Juan D. Carvajal-Agudelo, Tamara A. Franz-Odendaal

**Affiliations:** Department of Biology, Mount Saint Vincent University, Halifax, NS, Canada

**Keywords:** bone homeostasis, microgravity, osteoclast, osteoclastogenesis, skeleton, zebrafish

## Abstract

**Introduction:**

Microgravity is one type of external stimulus that affects bone homeostasis and bone development. This study investigates the molecular drivers of these effects in order to more fully understand the cellular communication network between bone cells when bone homeostasis is perturbed.

**Methods:**

The transcriptional responses of bone-related genes in zebrafish larvae (*Danio rerio*) when exposed to simulated microgravity (SMG) using a Random Positioning Machine were analysed. Larvae were initially analyzed at 6, 12, 18, and 24 h post-exposure via RT-qPCR with a focus on the RANKL/RANK/OPG pathway.

**Results:**

Short exposures (6–12 h) produced minimal changes, whereas 18–24 h SMG triggered a two-phase response: initial suppression of osteoblast markers (*bglap, sp7, alpl*, collagens) followed by activation of osteoclast-associated genes (*tnfsf11*/RANKL, *tnfrsf11b*/OPG, *tnfrsf11a*/RANK, *nfatc1, ctsk*) and stress-adaptive pathways (*hsp* family). We then conducted a transcriptomic analysis at 18 and 24 h. Transcriptomic and gene–protein interaction network analyses revealed distinct regulatory clusters encompassing extracellular matrix and osteoclast signaling genes, highlighting the coordinated modulation of bone formation and resorption. Functional enrichment analyses confirmed the involvement of WNT, BMP, HIPPO, and MAPK signaling pathways in skeletal regulation under SMG, and activated stress-adaptive pathways while concurrently downregulating apoptosis-related genes reflecting a complex interplay among developmental, metabolic, and disease-associated bone processes.

**Discussion:**

This data highlights a developmental stage-specific protective response. Collectively, these results demonstrate that SMG disrupts the balance between osteoblast and osteoclast activity, promoting bone resorption via the RANKL/RANK/OPG pathway while suppressing matrix deposition. These findings lay the groundwork for designing targeted interventions to mitigate bone loss during spaceflight and in osteoporotic conditions.

## Introduction

In the absence of normal gravitational loading, organisms undergo physiological changes such as bone loss, muscle atrophy, and altered cellular signaling (Corydon et al., 2023; Bizzarri et al., 2015). Understanding these effects is critical for developing strategies to mitigate health risks associated with long-term spaceflight, and other bone-related diseases such as osteoporosis (Baran et al., 2022). Several studies have shown that the bone loss documented in astronauts resembles osteoporosis, with the pathology progressing more quickly with exposure to microgravity (Nagaraja and Risin, 2013; Man et al., 2022) Ground-based laboratories use simulated microgravity (SMG) systems, with a preference for clinostats and random positioning machines (RPM) to simulate microgravity environments (Calvaruso et al., 2021; Eaton et al., 2023) and to induce an osteoporosis-like phenotype in animal models. These machines allow researchers to investigate how SMG influences cellular processes, gene expression patterns, and tissue development in controlled environments.

As research into the impact of microgravity on living organisms continues to grow, zebrafish (*Danio rerio*) have emerged as a powerful vertebrate model organism in biology due to their genetic similarity to humans, transparent embryos, and rapid development (Dietrich et al., 2020). In particular, zebrafish have become an excellent model to study bone homeostasis due to the similarity in bone deposition and resorption mechanisms with humans (Aceto et al., 2016; Barua et al., 2021; Deng et al., 2017; Kim et al., 2013; Carnovali et al., 2016; Pasqualetti et al., 2012; Li et al., 2009). Research into SMG exposure using zebrafish has revealed insights such as decreased bone density and reduced bone formation in larvae and long-term skeletal abnormalities in adult zebrafish (Eaton et al., 2023; Aceto et al., 2016). It is also associated with developmental impairments, including low hatching rates and high mortality rates likely due to increased larval malformations (Gillette-Ferguson et al., 2003; Li et al., 2020; Franz-Odendaal and Edsall, 2018).

Studies on gene expression and transcriptomics in zebrafish under SMG conditions have provided valuable insights into the biological impact of altered gravity on development. Notable findings include disruptions in gene pathways related to bone, muscle, and cardiovascular systems (Gillette-Ferguson et al., 2003), decreased p53 expression in embryos (Hang et al., 2011), and upregulation of genes involved in cell proliferation, differentiation (Lien, 2022), DNA repair, and metabolism (Barua et al., 2021). Specifically, bone studies have shown that, after 24 h of simulated microgravity exposure, the number of *Runx2a* expressing cells, a marker for osteoblasts, decreased in adult zebrafish (Carvajal-Agudelo et al., 2023) while larvae exhibited reduced overall ossification and increased *tnfsf11* (RANKL) expression (Carvajal-Agudelo et al., 2024). In this study, we explore gene differential expression related to bone development in larval zebrafish exposed to simulated microgravity to add a deeper understanding of the molecular response to SMG in early developmental stages. These investigations provide valuable insights into the molecular mechanisms driving organismal and bone tissue responses to altered gravitational forces with implications for osteoporosis and related bone pathologies. These findings not only highlight potential health risks associated with prolonged microgravity exposure but also have broader implications for human health, including the development of treatments for bone loss and other cellular dysfunctions.

## Results

### 18 h and 24 h SMG exposure reveals a critical window for modulation of RANKL/RANK/OPG signaling pathway

**FIGURE 1:**
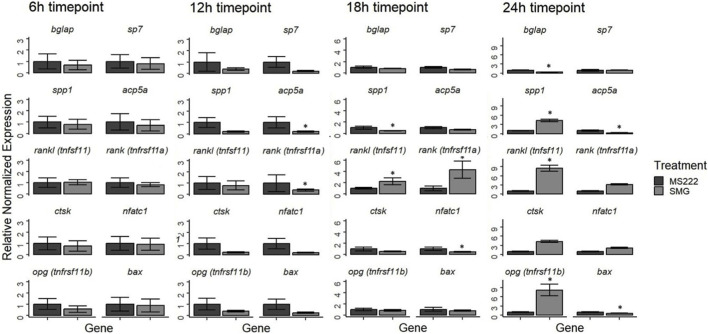
Relative normalized gene expression of selected genes measured by RT-qPCR at 6, 12, 18, and 24 h of simulated microgravity (SMG) exposure. Expression values were normalized to the housekeeping genes *β-actin* and *rpl13a* and calculated relative to the untreated control (MS-222). Bars represent mean ± standard error of the mean (SEM) from three biological replicates, and asterisks (*) indicate statistically significant differences compared to control (p < 0.05).

**FIGURE 2:**
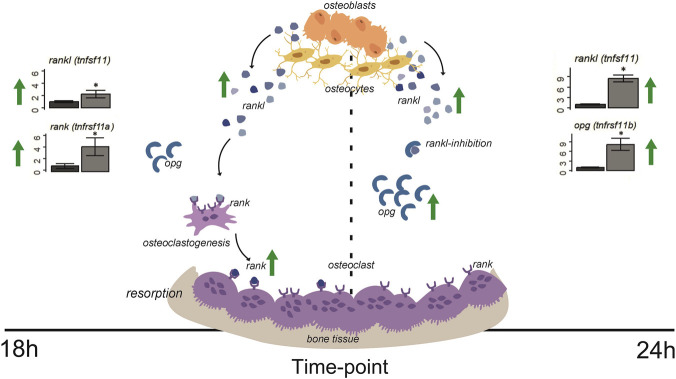
The RANKL/RANK/OPG signaling pathway and its temporal gene expression dynamics under SMG exposure. This diagram depicts the RANKL/RANK/OPG signaling axis, where RANKL binds to rank receptors on osteoclast precursors to promote their differentiation into mature osteoclasts. *opg* acts as a decoy receptor for RANKL, inhibiting osteoclastogenesis. The figure includes the study window timeline showing how SMG influences the expression of these genes over time, highlighting potential alterations in osteoclast activity and bone resorption under SMG. Image was partially designed using BioRender.

Gene expression patterns varied depending on the SMG exposure duration. At 6 h, no significant changes were observed across any of the analyzed genes, indicating a stable gene expression profile after a short exposure to SMG (Figure 1). By 12 h, *acp5a*, a marker of osteoclast activity, and *tnfrsf11a* (RANK) were significantly downregulated, suggesting an early suppression of osteoclast-related signaling. At 18 h, *spp1* and *nfatc1*, both associated with osteoclast differentiation and function, were significantly decreased, while *tnfrsf11a* (RANK) and *tnfsf11* (RANKL) were significantly upregulated, pointing to a shift toward enhanced osteoclast signaling despite reduced expression of some downstream regulators (*spp1* and *nfatc1*). This time point marked a notable shift in the RANKL/RANK/OPG system (Figure 2), with an increase in both receptor (RANK) and ligand (RANKL). At 24 h *bglap* (osteoblast marker), *acp5a*, and *bax* (apoptosis-related) were significantly downregulated, while *spp1*, *tnfsf11* (RANKL), and *tnfrsf11b (opg)* were significantly upregulated (Figure 1). Taken together, these results indicate that SMG exposure induces a time-dependent modulation of bone-related gene expression, followed by the regulation of RANKL/RANK/OPG signaling components (Figure 2). The simultaneous increase of both RANKL and its decoy receptor *opg* indicates a dynamic modulation of this pathway at prolonged exposures. Overall, these results reveal that SMG exposure induces a time-dependent transcriptional response, with early suppression of bone markers followed by selective activation of the RANKL/RANK/OPG signaling pathway, a key regulator of osteoclastogenesis and bone remodeling.

### Transcriptomic analysis highlights suppressed osteoblast activity and enhanced signaling

**FIGURE 3:**
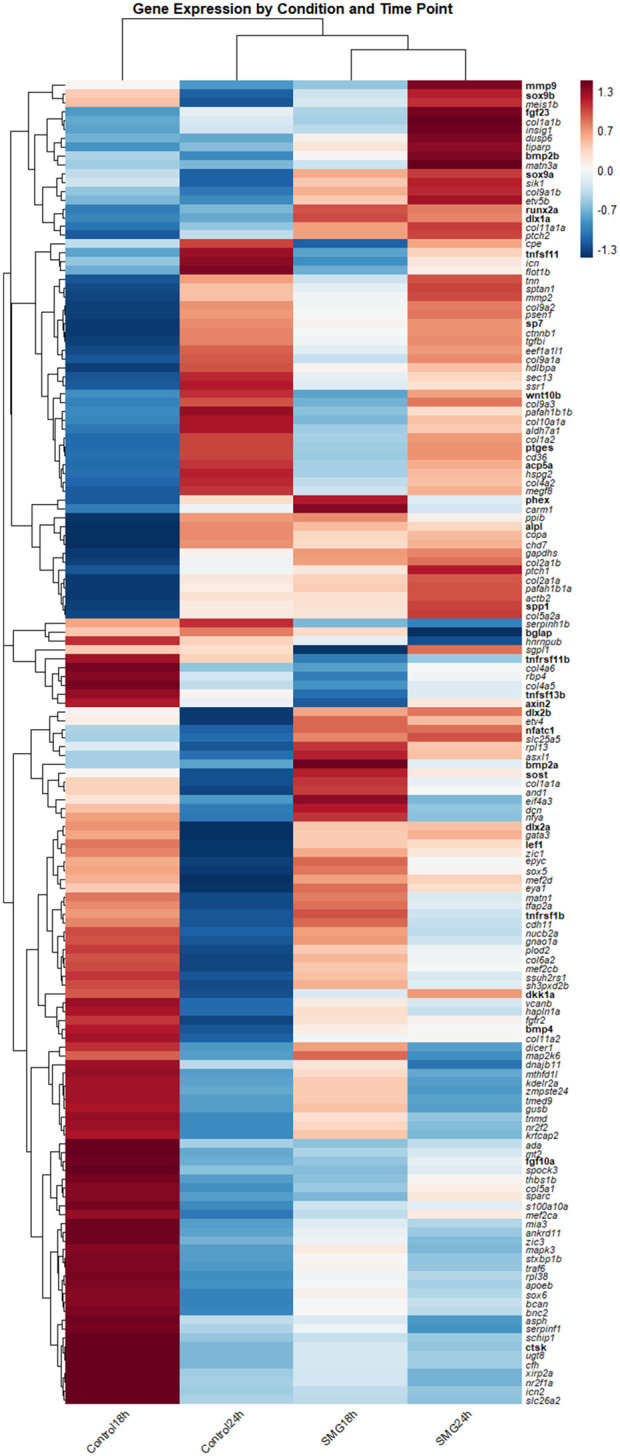
Heatmap of transcriptomic results showing the expression profiles of selected bone-related genes at 18 and 24 h after SMG exposure. The first two columns show the control groups and the second two columns show the treatment groups. Expression levels are represented as normalized TPM values across biological replicates. Hierarchical clustering was applied to both genes and samples to highlight patterns of up- and downregulation over time. The color scale indicates relative expression, with higher expression in red and lower expression in blue allowing visualization of temporal changes in gene expression in response to SMG. Shades of red (e.g., pink) and blue (e.g., light blue) reflect expression levels close to the high or low levels respectively.

Transcriptome analysis revealed clear time-dependent transcriptional shifts in bone-related pathways under SMG (Figure 3). At 18 h, SMG exposure induced clusters of changes in genes showing distinct up- or downregulation compared to controls. Several osteoclast-associated genes, including *ctsk, mmp9* and components of the RANKL/RANK/OPG system, were strongly upregulated (*tnfrsf11b/*OPG and *tnfsf11/*RANKL), and downregulated (*tnfrsf11a/*RANK) suggesting early activation of resorption-related signaling (Figure 3). In contrast, osteoblast markers such as *alpl, bglap, nfatc1* and *spp1* and matrix-associated collagens *(col1a1a, col6a2 col2a1a, col2a1b, col9a1b, col11a1a)* were suppressed, reflecting a reduction in matrix deposition and osteoblast activity.

By 24 h, the transcriptional response intensified. The RANKL/RANK/OPG pathway became more prominent, *opg* (*tnfrsf11b*) and RANK (*tnfrsf11a*) are induced while *tnfsf11/*RANKL is supressed, and downstream osteoclast regulators, including *ctsk, traf6, and mmp9*, remained elevated. In parallel, osteoblast-associated genes such as *bglap, alpl, col10a1a,* several extracellular matrix collagen structural genes (*col1a1b, col2a1a, col9a1b*) continued to be repressed (Figure 3). This indicates a persistent shift toward osteoclast signaling and a weakening of osteoblast differentiation and matrix mineralization. Osteocyte-associated regulators also showed altered expression, *sost* and *phex* were supressed and then upregulated under SMG after 18h, suggesting a shift in mechanosensory signaling that could further suppresses osteoblast-driven bone formation.

Beyond direct bone markers, several apoptosis and stress-responsive genes were differentially expressed. Pro-apoptotic regulators such as *bax* were downregulated, while stress adaptation pathways, including members of the *hsp* family, were upregulated after 24 h, suggesting a protective response or a countermeasure to transcriptional responses. Some other key bone related developmental and signaling pathways also showed modulation: *wnt10b*, *fgf10a,* were induced, while inhibitors such as *dkk1a* and transcription factors including *nfatc1, sox9a* and *sox9b* were reduced.

Taken together, the transcriptome data indicates that SMG triggers a two-phase response: an early transcriptional reorganization at 18 h marked by the first activation of osteoclast signaling, followed by a more robust response at 24 h characterized by strong induction of the RANKL/RANK/OPG system and persistent suppression of osteoblast markers and extracellular matrix deposition. This result aligns with the analysis of DEGs under SMG exposure, which revealed that both upregulated and downregulated significant genes were largely time-specific as reflected by the few genes shared across time points (Supplementary Material 3).

### Gene–protein network reveals expression-based clusters of bone structure and remodeling

**FIGURE 4:**
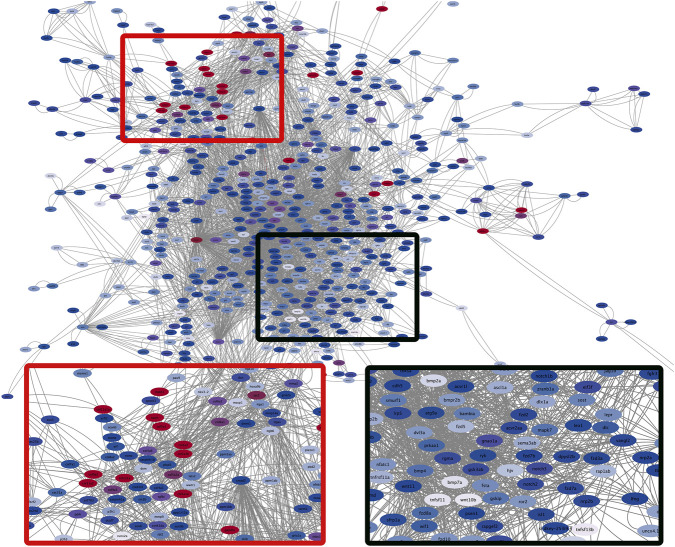
Bone gene interaction network at 24 h of SMG. The network was constructed using STRING protein–protein interaction data and visualized in Cytoscape. Node size reflects the correlation of expression with other network genes, highlighting highly co-expressed genes with larger nodes. Node color indicates differential expression after 24 h of SMG: red nodes are upregulated, blue nodes are downregulated. Shades of red (i.e., purple) and of blue (i.e., grey) indicate genes that are slightly upregulated or slightly downregulated, respectively.

The gene network (Figure 4) shows the relationships among bone-related genes at 24 h SMG, highlighting co-expression connections. We observed two main clusters. One cluster in the 24 h SMG bone gene network is dominated by collagen and extracellular matrix genes, including *col11a2, col2a1a, col1a1a, col1a2, col5a1, col9a2, col10a1a, col9a3, col4a6, col5a2a*, as well as *sparc, matn1, tgfbi*, and *csnk1da*. These genes form a module related to bone matrix structure and organization. The high expression of this cluster indicates a tightly connected network of structural components critical for maintaining bone integrity and extracellular matrix assembly, reflecting the coordinated regulation of matrix genes under 24 h SMG conditions. This cluster likely corresponds to the highly expressed collagen and extracellular matrix genes observed in the heatmap, showing a coherent module of bone structural genes.

The other cluster consists of genes with lower expression levels, including *tnfsf11* (RANKL), *tnfrsf11a* (RANK)*, wnt10b, bmp7a, nfatc1, bmp4, bmp2a, bmp2b, dkk1a, tnfsf13b, and dlx0a*. These genes are primarily involved in osteoclast differentiation, bone signaling pathways, and developmental regulation. The reduced expression of this cluster suggests a distinct regulatory module within the network, highlighting genes that may coordinate signaling for osteoclastogenesis and bone remodeling. The presence of RANKL and RANK indicates potential modulation of osteoclast activation, and the network patterns here correspond to lower-expression regions in the heatmap, showing that while structural extracellular matrix genes remain strongly expressed, key bone related signaling and regulatory components are selectively downregulated.

### Functional enrichment of bone-associated genes reveals core pathways in skeletal development and signaling

**FIGURE 5:**
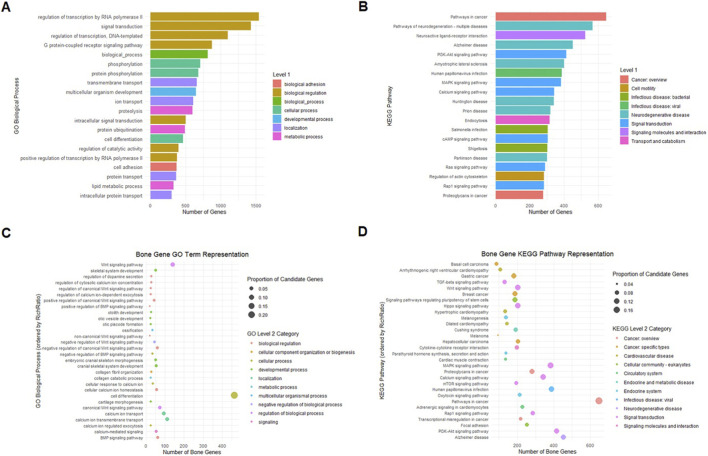
This figure summarizes the results of functional enrichment analyses for transcriptomic data collected at 18 h and 24 h after SMG treatment. Panels A and B show the results for all expressed genes. **(A)** presents Gene Ontology (GO) enrichment analysis, categorizing genes into biological processes, molecular functions, and cellular components, while **(B)** shows KEGG pathway enrichment analysis. Panels **(C,D)** focus on a subset of genes related to bone biology: C presents GO enrichment, and D shows KEGG pathway enrichment. Bubble sizes indicate either the number of genes associated with each term/pathway or the proportion of candidate genes, and colors represent Level 2 categories in GO or KEGG, emphasizing key functional and signaling pathways. Each panel has its own color key.

The enrichment analysis of bone-associated genes, filtered by RichRatio, revealed pathways and biological processes that, while representing a relatively small proportion of the overall candidate genes, highlight critical regulators of skeletal biology (Figure 5). At the general level, enrichment analyses of all expressed genes pointed toward broad processes such as transcriptional regulation, signal transduction, and stress-related pathways (e.g., MAPK and PI3K-Akt signaling, neurodegeneration-related networks). However, when narrowed to the bone-associated set, the KEGG pathway enrichment revealed strong representation in signaling pathways that are central to skeletal regulation and remodeling. Prominent pathways included WNT signaling, TGF-β signaling, HIPPO signaling, and MAPK signaling, all of which are recognized regulators of osteoblast differentiation, osteoclast activity, and bone matrix dynamics (Aman et al., 2018; Yang et al., 2015; Attisano and Wrana, 2013). Several pathways also overlapped with cancer and metabolic disease categories (e.g., proteoglycans in cancer, PI3K-Akt signaling pathway, mTOR signaling), reflecting the shared molecular mechanisms between bone remodeling and pathological cell proliferation (Figure 5B). Cardiovascular and endocrine-related pathways (such as calcium signaling and parathyroid hormone synthesis and secretion) were also significantly enriched, highlighting the systemic regulation of bone homeostasis. Overall, the KEGG network emphasizes the interconnection of bone gene signaling, spanning developmental, metabolic, and disease-related processes.

The GO enrichment analysis of bone-associated genes revealed a strong focus on developmental and signaling processes relevant to skeletal biology (Figures 5C,D). Key enriched terms included skeletal system development, cranial skeletal system morphogenesis, and cartilage morphogenesis, directly linking the transcriptomic data to bone formation and structural organization. Signaling-related processes such as the WNT signaling pathway (canonical, non-canonical, and positive regulation), BMP signaling pathway, and regulation of calcium ion homeostasis were also highly enriched, underscoring the importance of these signaling cascades in regulating bone gene function. Processes tied to extracellular matrix remodeling, such as collagen fibril organization and collagen catabolic process, were also represented, consistent with structural regulation of bone tissue. Together, these results demonstrate that bone-related genes under SMG map strongly to pathways and processes that govern development, extracellular matrix organization, and key signaling axes (WNT, BMP, calcium regulation) that underlie bone homeostasis.

## Discussion

This study demonstrates that SMG exposure induces dynamic and time-dependent transcriptional changes in zebrafish larvae, particularly within bone-related pathways. Based on the gene expression changes in the heatmap (Figure 3), we observed an early suppression of osteoblast-associated markers (e.g., *bglap*, *sp7*, *alpl*, *col1a1a*), accompanied by a progressive activation of osteoclast signaling through the RANKL/RANK/OPG system. Following the qPCR gene expression data (Figure 1), by 24 h, the simultaneous upregulation of *tnfsf11* (RANKL) and its decoy receptor *tnfrsf11b* (*opg*) highlights the activity of the RANKL/RANK/OPG pathway related to bone resorption regulation under SMG (Figure 2), suggesting the existence of a compensatory mechanism to balance osteoclast activation while osteoblasts are suppressed, thereby attempting to maintain bone homeostasis over time. These findings are consistent with prior studies showing that bone resorption is favored under microgravity while bone formation is impaired (Eaton et al., 2023; Aceto et al., 2016; Carvajal-Agudelo et al., 2023). Notably, some investigations suggest that the skeletal response may vary depending on developmental stage, exposure duration, and species, indicating that adaptive or compensatory mechanisms may coexist alongside resorptive pathways (Baran et al., 2022; Chatziravdeli et al., 2019; Prasad et al., 2020).

The RANKL/RANK/OPG signaling pathway has been described as a central regulatory axis of bone remodeling in vertebrates, mediating osteoclast differentiation and activity, with RANKL promoting bone resorption through its interaction with RANK, and *opg* functioning as a soluble decoy receptor that inhibits this process (Boyle et al., 2003; Theoleyre et al., 2004; Silva and Branco, 2011; Lacey et al., 2012; Kim et al., 2022). This system is particularly relevant in conditions of skeletal unloading such as microgravity, for example, spaceflight and simulated microgravity studies have consistently shown an upregulation of RANKL expression accompanied by a reduction in *opg*, leading to enhanced osteoclast activity and bone resorption (Chatziravdeli et al., 2019; Rucci et al., 2007; Liu et al., 2022). This dysregulation represents a critical mechanism driving microgravity-induced bone loss, underscoring both the evolutionary conservation of the RANKL/RANK/OPG axis and its pronounced responsiveness to mechanical unloading. Importantly, similar molecular patterns are observed in osteoporosis and other metabolic bone diseases, suggesting that microgravity serves as a valuable model to study the fundamental mechanisms underlying pathological bone loss (Hofbauer et al., 2004; Bai et al., 2011; Dougall and Chaisson, 2006).

Other regulatory genes like *nfatc1* and cathepsin K (*ctsk*), play pivotal roles in osteoclast differentiation and function. *nfatc1*, a master regulator of osteoclastogenesis, is essential for the differentiation and activation of osteoclasts (Silva and Branco, 2011; Boyce and Xing, 2007; Udagawa et al., 2021). It mediates osteoclast fusion and activation by upregulating genes responsible for osteoclast adhesion, migration, acidification, and degradation of both inorganic and organic bone matrix components (Silva and Branco, 2011; Udagawa et al., 2021; Kawai et al., 2023). Similarly, *ctsk*, a lysosomal cysteine protease predominantly expressed in osteoclasts, is crucial for bone resorption. It degrades bone matrix proteins such as type I collagen, osteopontin, and osteonectin, facilitating the breakdown of bone and cartilage (Kawai et al., 2023; Lotinun et al., 2013). Together, *nfatc1* and *ctsk* orchestrate the complex processes of osteoclast differentiation and bone resorption, highlighting their significance in skeletal biology. Abnormal regulation of these genes has been directly implicated in pathological conditions such as osteoporosis and other osteolytic bone diseases (osteoporosis and rheumatoid arthritis), where excessive osteoclast activity disrupts bone homeostasis and leads to progressive bone loss (Novack and Teitelbaum, 2008; Drake et al., 2015; Takayanagi, 2007).

The transcriptome analysis also revealed that SMG exposure not only impacts direct bone markers but also modulates signaling pathways central to skeletal homeostasis, including WNT, BMP, and MAPK signaling. The repression of osteoblast transcription factors such as *sox9* and structural extracellular matrix collagens indicates that matrix formation and mineralization are suppressed early in the response (Figures 3, 4). At the same time, osteocyte-associated genes such as *sost*, *fgf23*, and *phex* suggests altered mechanosensory signaling, consistent with reduced loading environments where osteocytes act to inhibit new bone deposition (Beck-Nielsen et al., 2019; Sebastian and Loots, 2017; Fukumoto, 2019; Quarles, 2003). These transcriptomic signatures strongly support the hypothesis that SMG disrupts the balance between osteoblast and osteoclast activity, favoring bone resorption. This result also aligns with the network analyses that revealed two distinct regulatory clusters: (1) a cluster dominated by collagens and extracellular matrix genes, and (2) a signaling cluster centered on RANKL, RANK, WNT10B, and BMP family members (Figure 4). The coexistence of these clusters highlights the coordinated but opposing regulation of bone matrix stability and resorptive signaling under SMG. Importantly, enrichment of pathways such as WNT, HIPPO, and TGF-β signaling confirms the broad systemic impact of SMG on skeletal development and regulation, (Yang et al., 2015; Attisano and Wrana, 2013; Erlebacher et al., 1998; Tyrina et al., 2024). These pathways are known to be activated following activation of osteocytes by mechanical force alongside the RANKL/RANK/OPG system (Sasaki et al., 2021). For example, the HIPPO pathway can regulate bone-related cells through its own signaling pathway or via bone-related signaling pathways such as the Wnt pathway, the Notch pathway and the RANKL pathway (Shao et al., 2025). Furthermore, dysregulation of these pathways are also implicated in bone diseases such as osteoporosis, osteopetrosis, and other metabolic bone disorders (Baron and Kneissel, 2013; Baron et al., 2011; Krishnan et al., 2006). Together, these findings demonstrate that SMG induces a dual regulatory system: downregulation of bone formation components while simultaneously activating osteoclast-promoting signals, resulting in imbalanced bone remodeling similar to the molecular mechanisms underlying osteoporosis (Krishnan et al., 2006; Brömme and Lecaille, 2009). The network analysis not only confirms the transcriptomic signatures but also identifies key regulatory nodes and potential therapeutic targets for mitigating bone loss during spaceflight or in osteoporotic bone loss conditions.

The temporal dynamics observed in this study suggest that the transcriptional response to SMG is not immediate but instead develops progressively over time. SMG could induce immediate cellular changes that are not yet detectable at the transcriptional level within the first 6–12 h, either due to buffering mechanisms or the sensitivity limits of qPCR detection. However, the coordinated shift observed at 18–24 h supports that sustained mechanical unloading is required before a clear transcriptional change is initiated. In bone biology, mechanosensitive cells particularly osteocytes are known to integrate mechanical signals over time before activating downstream remodeling pathways (Bonewald, 2011; Robling et al., 2006). This concept aligns with the classical mechanostat model, in which bone responds once a threshold of altered mechanical strain is reached rather than reacting instantaneously (Frost, 1987). The delayed activation of the RANKL/RANK/OPG axis observed here may therefore reflect a cumulative response to continued unloading, consistent with the two-phase pattern in which early suppression of osteoblast markers precedes the activation of osteoclast-related signaling.

Under normal conditions, the zebrafish musculoskeletal system is continuously exposed to mechanical loading generated by gravity, buoyancy, and locomotor muscle contraction, which together produce internal stresses and strains in bone and muscle (Franz-Odendaal and Edsall, 2018; Kitamura et al., 2013). In our RPM-based SMG paradigm, the gravity vector is continuously reoriented, reducing consistent directional loading and diminishing sustained strain signals that are normally detected by mechanosensitive cells (Calvaruso et al., 2021; Eaton et al., 2023). We propose that this reduction in effective mechanical input is integrated over time at the tissue level: (i) decreased locomotor-driven forces and altered hydrostatic/fluid mechanical cues reduce bone strain and mechanotransduction (Bonewald, 2011; Robling et al., 2006), (ii) osteocytes and osteoblast-lineage cells respond by shifting signaling toward an “unloading” program (including changes in osteocyte-associated regulators and suppression of osteoblast/matrix markers) (Chatziravdeli et al., 2019; Rucci et al., 2007; Liu et al., 2022), and (iii) this precedes a secondary remodeling-like response in which osteoclast-related signaling is promoted through the RANKL/RANK/OPG axis (Boyle et al., 2003; Theoleyre et al., 2004; Silva and Branco, 2011; Lacey et al., 2012; Kim et al., 2022). In this framework, the delayed transcriptional shift (18–24 h) reflects the time required for reduced strain to accumulate and for mechanosensory pathways to reach a threshold that triggers coordinated remodeling responses rather than an instantaneous reaction to altered gravity (Robling et al., 2006; Frost, 1987; Liu et al., 2023).

Although we did not directly quantify tissue stresses/strains in this study, this mechanobiological model provides an explanation for the observed two-phase response, where early suppression of osteoblast and extracellular matrix programs is followed by activation of osteoclast-associated signaling. Future work combining imaging-based assays (e.g., larval skeletal confocal markers), muscle activity proxies, and other tissue markers that measure physiological changes would allow explicit estimation of strain distributions under SMG and direct testing of this tissue-level model.

Interestingly, apoptosis-related genes (*bax*) were downregulated, while stress-response and protective pathways (*hsp* family genes) were induced after 18 h (Figure 1 for *bax*; Figure 3). This finding contrasts with reports of increased cell death in both mammalian and zebrafish models under SMG (Corydon et al., 2023; Hang et al., 2011; Carvajal-Agudelo et al., 2023; Prasad et al., 2020; Grimm et al., 2002; Riwaldt et al., 2021). In the same way, the modulation of apoptosis and stress-response pathways may reflect a compensatory mechanism to preserve osteoblasts while under mechanical strain, and its dysregulation could contribute to imbalanced bone remodeling observed in osteoporosis and other bone-loss disorders (Huang et al., 2023; Wiren et al., 2006). These latter zebrafish studies used adult zebrafish bone (i.e., scales). It is possible that zebrafish larvae exhibit stage-specific compensatory mechanisms that mitigate SMG-induced stress. In support of this, the general KEGG enrichment revealed pathways such as PI3K-Akt, MAPK, and calcium signaling, which are directly linked to cell survival, apoptosis regulation, and adaptive stress responses (Pinton et al., 2008; Franke et al., 2003; Yue and López, 2020). Additionally, enrichment of neurodegeneration-associated pathways (Alzheimer, Parkinson, Huntington, ALS) highlights molecular processes tied to oxidative stress and programmed cell death, while cancer-related pathways (e.g., proteoglycans in cancer, PI3K-Akt) reflect the overlap between pro-survival and proliferative signaling (Figure 5). Our results consistently highlight the upregulation of stress-protective pathways across multiple replicates, while pro-apoptotic markers remained suppressed, pointing toward an adaptive rather than degenerative response at this developmental stage. These findings suggest that while microgravity induces stress, both zebrafish larvae and mammals may activate conserved protective mechanisms to counteract potential cell death (Prasad et al., 2020; Uva et al., 2002; López Garzón et al., 2025; Cubbage and Mabee, 1996). The differential responses observed across species highlight the complexity of cellular adaptations and underscore the need for further comparative studies to elucidate the underlying mechanisms.

In summary, our results show that SMG rapidly alters the expression of key genes regulating bone homeostasis in zebrafish, particularly by suppressing osteoblast activity and enhancing osteoclast signaling via the RANKL/RANK/OPG pathway. Although our findings are consistent with a shift toward osteoclast-promoting signaling under SMG, the observed stress-adaptive and anti-apoptotic signatures suggest that larvae may also engage protective mechanisms, highlighting skeletal responses to unloading effects (Corydon et al., 2023; Krishnan et al., 2006). It is important to note that microgravity does not uniformly suppress bone formation across all settings. Instead, responses appear to depend on the balance between mechanical input, developmental timing and systemic signaling, highlighting the complexity of skeletal adaptation rather than an entirely degenerative process (Bonewald, 2011; Robling et al., 2006; Riwaldt et al., 2021). Our findings not only reinforce the zebrafish as a valuable model for studying the skeletal effects of microgravity but also provide a foundation for identifying molecular targets for countermeasures against spaceflight-induced bone loss as well as for bone loss disorders such as osteoporosis.

Future studies should extend these experiments to longer time exposure treatments, assess recovery following SMG, integrate other bioinformatics approaches and validate outcomes through functional methods (e.g., bone density measurements, histological assays) to confirm the physiological impact of the observed gene expression changes.

## Materials and methods

### Ethics statement

All protocols follow the Canadian Council on Animal Care (CCAC) guidelines and were approved by the Saint’s Mary University and Mount Saint Vincent University Animal Care Committee under the protocol numbers 21–10, 22–03, 23-13 and 24-12.

### Zebrafish maintenance and sample preparation

Wildtype zebrafish (*D. rerio*) were used for all experiments and embryos were raised in the Mount Saint Vincent University fish facility according to standard procedures (28.5 °C, 12–12-h light cycle). All larvae used were obtained from the Zebrafish Core Facility at the Life Science Research Institute, Dalhousie University. Zebrafish at 3.5 dpf (between 3.0 and 3.5 mm SL) were randomly divided into groups of no more than ten fish and placed in 50 mL falcon tubes with zebrafish rearing water. This age was selected because several bones begin to ossify between 3-4 dpf based on whole-mount staining of the skeleton (Eaton et al., 2023; Cubbage and Mabee, 1996) and the fish are not actively feeding yet. It is well recognised that earlier detection of skeletal elements is possible using alternative methods (such as histology (Clark and Smith, 1993)), and that gene expression precedes overt differentiation of the skeleton with some markers (e.g., *Runx2a/b*) expressed as early as 36 hpf in cranial skeletal elements (Li et al., 2009). In order to ensure the larvae could not swim during exposure, an anaesthetic (0.01% MS222) was added to the water of all treatment groups. Methylene blue 1% (Sigma Aldrich) at a concentration of 0.1 μL/10 mL was also added to all treatment groups. The water with the addition of the anaesthetic and methylene blue constitutes the media. Control untreated samples and control contained MS222 were raised in a separate incubator and were reared at the same time.

### Exposure to simulated microgravity

Simulated microgravity was generated using a RPM (Random Positioning Machine), obtained from YURI (Meckenbeuren, Germany). The RPM was set to rotate in real random mode with a maximum rotational speed of 65° per second, and a minimum of 53° per second. According to previous protocols (Eaton et al., 2023; Carvajal-Agudelo et al., 2024); these parameters were selected based on optimisation experiments that demonstrated that the fish survive under this state of rotation during treatment.

The falcon tube was positioned on the center of the RPM platform. The experiment included four time points for RT-qPCR: exposure for 6 h, 12 h, 18 h, and 24 h, and for transcriptomics exposure only 18 h, and 24 h were analyzed. These latter time points were selected based on the qPCR results. All fish were 3.5 dpf (3.0–3.5 mm SL) at the start of the experiment. At this timepoint zebrafish exhibit early skeletal development, actively developing and undergoing first stages of ossification and cartilage development (Riwaldt et al., 2021). At the end of each time point, a subset of fish was collected. Control groups included untreated samples, and those with the addition of MS222. After the experiments were concluded, all fish were euthanized using an overdose of buffered MS222 (0.1%). Samples for RT-qPCR analyses were immediately stored in *RNA Later* solution (Invitrogen) at −20 °C.

### Gene expression RT-qPCR base analyses

Samples were pooled with 30 larvae per biological replicate for a total of three biological replicates (90 larvae per group). Total RNA was isolated using RNeasy Plus Universal Mini Kit (Qiagen). RNA quality was evaluated via a 1% agarose gel electrophoresis and quantified with a BioDrop (Montreal Biotech Inc.). Primers were derived from the literature (Table 1) and a melt curve analysis was performed to confirm primer specificity. The cDNA dilution factor was optimized for each primer pair using a standard curve, and only primers with a reaction efficiency of 90%–110% were used. cDNA syntheses were performed using the iScript™ Reverse Transcription Supermix (BioRad Laboratories). Each reaction contained 300 nM primers, 5 μL SsoAdvanced Universal SYBR Green Supermix (Bio-Rad Laboratories) and 4 μL of cDNA. The amplifications were performed on a CFX384 Touch Real-Time PCR Detection System (Bio-Rad Laboratories) according to the following thermal conditions: initial denaturalization 95 °C for 2 min followed by 40 cycles of amplification, consisting of denaturation at 95 °C for 10 s, annealing at 60 °C for 20 s and extension at 72 °C for 20 s. Secondary denaturation was then performed at 95 °C for 10 s followed by a melt curve analysis from 65 °C to 95 °C in 0.5 °C increments for 5 s each. Three biological replicates and at least two technical replicates in addition to a no template control and no transcription controls were included.

**TABLE 1:** Primer sequences and conditions used in the current study.

Gene	Strand	Sequence (5′–3′)	References
*acp5a*	F	CCA​TGT​AGG​AAA​CGT​CAA​AGC	Han et al. (2021)
	R	GAA​TGC​GGA​AGT​TCA​TCT​CAT	
*actb1*	F	TTA​CCA​CTT​CAC​GCC​GAC​TC	Xu et al. (2018)
	R	GTC ACCTTCACCGTTCCAGT	
*bax*	F	GGC​TAT​TTC​AAC​CAG​GGT​TCC	Lerebours et al. (2009)
	R	TGC​GAA​TCA​CCA​ATG​CTG​T	
*bglap*	F	CTG​CTG​CCT​GAT​GAC​TGT​GT	Chen et al. (2019)
	R	CAC​GCT​TCA​CAA​ACA​CAC​CT	
*ctsk*	F	ATG​ATC​TGG​GCA​TGA​ACC​AT	Kitamura et al. (2013)
	R	CCG​AAG​TGA​CGT​ATC​CCA​GT	
*nfatc1*	F	AAC​CTT​CCT​CGT​TCC​CTC​AA	Kitamura et al. (2013)
	R	CGC​TGT​TAT​CCT​CCA​CCT​CA	
*rpl13a*	F	CCC​TTC​CCG​TGG​ATC​ATA​TC	Wang et al. (2019)
	R	TTT​GCG​TGT​GGG​TTT​CAG​AC	
*spp1*	F	CAT​GAT​CTG​GAG​GAC​GGG​AA	He et al. (2018)
	R	CTC​TTC​TGT​AGC​TGC​CTG​GT	
*sp7*	F	TAA​ACC​GGG​AAG​CAC​CAT​CC	Niu et al. (2017)
	R	AAG​AAG​ACG​TGG​CGT​TAG​CA	
*tnfsf11 (*RANKL)	F	CTC​ACC​TTC​CAA​TCA​AGA​CGC​CC	Ahi et al. (2016)
	R	CTT​TCA​TGC​CAT​CCC​AGG​CTA​TCT	
*tnfrsf11b (opg)*	F	GTC​AAA​ACC​GCT​GGA​ACG​CC	He et al. (2018)
	R	CAG​CAG​ATG​CTC​TTC​CCC​CTG	
*tnfrsf11a (*RANK)	F	AAG​TGG​ACA​GAT​TGT​AAA​GCT​AT	Carnovali et al. (2021)
	R	GCC​ACC​TGA​TGA​GGT​TTC​AGC​AC	

Relative normalized gene expression values were based on the methods described by (Wen, 2017). Gene expression values were calculated relative to each respective control. *actb1* and *rpl13a* were used as housekeeping genes (Table 1). A one-way ANOVA was performed with treatment as the independent variable. P < 0.05 was considered statistically significant in all analyses. Data were analyzed using the Biorad CFX Maestro v1.0.

### Transcriptomics process and analysis

Samples were pooled with 30 larvae per biological replicate, resulting in three biological replicates per group (90 larvae total per group). RNA extraction, sequencing, and initial data analysis were performed by BGI Genomics (Hong Kong, China). Clean reads were mapped to the *D. rerio* reference genome (Ensembl release 105) using HISAT2 (Wen, 2017; Wang et al., 2024). Fusion genes and differential splicing events were detected with Ericscript (v0.5.5-5) and rMATS (v4.1.1), respectively (Wang et al., 2024). Gene expression levels were quantified with RSEM (v1.2.28) to obtain read counts, FPKM, and TPM values (Li and Dewey, 2011). Differential expression analysis was performed using DESeq2 (Love et al., 2014) (Poisson distribution, Q value ≤0.05). Initial gene annotation was performed using KEGG (v102.0), Gene Ontology (GO) databases (UniProt, NCBI Gene2GO, and GeneOncology idmapping), transcription factor and cofactor databases (AnimalTFDB v3.0, PlantTFDB), MsigDB (v7.1), GenBank, InterPro, Pfam, EggNOG, Reactome, CR2Cancer, and CellMarker. Missing KEGG pathway annotations were subsequently completed in a stepwise approach. Initially, KEGG pathways and gene identifiers were obtained from *D. rerio* using the Bioconductor package org. Dr.e.g.,.db for mapping Ensembl IDs to Entrez IDs and gene symbols, and the KEGGREST API to retrieve gene–pathway associations and pathway descriptions. For genes not mapped to KEGG pathways, human orthologs were identified using the Compara homology dataset and Ensembl BioMart, allowing retrieval of human gene symbols and IDs. KEGG enrichment analysis was then conducted on the human orthologs using the clusterProfiler package and org. Hs.e.g.,.db to identify relevant pathways. Enriched human KEGG pathways were subsequently mapped back to the zebrafish genes via the ortholog table, resulting in a comprehensive annotation for downstream analyses. Genes were then filtered based on KEGG pathways and GO terms to retain only those associated with bone-related processes (Supplementary Material 1). Additional genes related to bone tissue processes were incorporated from the mouse database Mouse Genome Database (MGD: *skeletal system development*), ZFIN database (*skeletal system*, *bone development, bone gene expression*), and selected publications (Ho et al., 2000; Chen et al., 2016). The following genes were manually added; *tnfsf11, tnfrsf11a, tnfrsf11b, bglap, acp5a, sp7, spp1, ctsk, nfatc1, tnfsf13b, runx2a, alpl, sost, ptges, dkk1a, phex, dlx1a, dlx2a, dlx2b, col1a1a, col1a1b, col1a2, mmp9, bmp2a, bmp2b, bmp4, axin2, lef1, wnt10b*, and *fgf23*. This resulted in a curated list of 2,350 bone-associated genes for downstream analyses which constitutes the bone gene database used in this analysis (Supplementary Material 2).

Gene expression analyses were performed to investigate transcriptional changes with a particular focus on bone-related pathways in zebrafish under experimental conditions. A heatmap was generated using TPMs and the normalized gene expression by DEGseq2 expression levels. The gene–protein interaction network was constructed using the gene expression data and by mapping genes to their corresponding protein identifiers using the STRING database (v11.5) in Cytoscape v. 3.10.3 for *D. rerio*, which provides the predicted protein–protein interactions (PPI). GO and KEGG enrichment results were filtered, and the top enriched terms were selected based on gene count or RichRatio to facilitate clearer visualization. Finally, differentially expressed genes (DEGs) and the bone gene database were compared across experimental conditions, and overlapping as well as unique gene sets were visualized using Venn diagrams.

## Data Availability

The raw data supporting the conclusions of this article will be made available by the authors, without undue reservation.

## References

[B1] Aceto J. Nourizadeh-Lillabadi R. Bradamante S. Maier J. A. Alestrom P. van Loon J. J. (2016). Effects of microgravity simulation on zebrafish transcriptomes and bone Physiology—Exposure starting at 5 days post fertilization. NPJ Microgravity 2, 1–8. 10.1038/npjmgrav.2016.10 28725727 PMC5515515

[B2] Ahi E. P. Walker B. S. Lassiter C. S. Jónsson Z. O. (2016). Investigation of the effects of estrogen on skeletal gene expression during zebrafish larval head development. PeerJ 4, e1878. 10.7717/peerj.1878 27069811 PMC4824909

[B3] Aman A. J. Fulbright A. N. Parichy D. M. (2018). Wnt/β-catenin regulates an ancient signaling network during zebrafish scale development. Elife 7, e37001. 10.7554/eLife.37001 30014845 PMC6072442

[B4] Attisano L. Wrana J. L. (2013). Signal integration in TGF-β, WNT, and hippo pathways. F1000Prime Rep. 5, 17. 10.12703/P5-17 23755364 PMC3672943

[B5] Bai P. Sun Y. Jin J. Hou J. Li R. Zhang Q. (2011). Disturbance of the OPG/RANK/RANKL pathway and systemic inflammation in COPD patients with emphysema and osteoporosis. Respir. Res. 12, 157. 10.1186/1465-9921-12-157 22176920 PMC3260206

[B6] Baran R. Wehland M. Schulz H. Heer M. Infanger M. Grimm D. (2022). Microgravity-related changes in bone density and treatment options: a systematic review. Int. J. Mol. Sci. 23, 8650. 10.3390/ijms23158650 35955775 PMC9369243

[B7] Baron R. Kneissel M. (2013). WNT signaling in bone homeostasis and disease: from human mutations to treatments. Nat. Med. 19, 179–192. 10.1038/nm.3074 23389618

[B8] Baron R. Ferrari S. Russell R. G. G. (2011). Denosumab and bisphosphonates: different mechanisms of action and effects. Bone 48, 677–692. 10.1016/j.bone.2010.11.020 21145999

[B9] Barua S. Komissarov A. Kaur H. Brodsky E. Transcriptomic analysis of DNA damage response in zebrafish embryos under simulated microgravity. (2021).

[B10] Beck-Nielsen S. S. Mughal Z. Haffner D. Nilsson O. Levtchenko E. Ariceta G. (2019). FGF23 and its role in X-linked hypophosphatemia-related morbidity. Orphanet J. Rare Dis. 14, 58. 10.1186/s13023-019-1014-8 30808384 PMC6390548

[B11] Bizzarri M. Monici M. van Loon J. J. W. A. (2015). How microgravity affects the biology of living systems. Biomed. Res. Int. 2015, 863075. 10.1155/2015/863075 25667927 PMC4312564

[B12] Bonewald L. F. (2011). The amazing osteocyte. J. Bone Mineral Research 26 (2), 229–238. 10.1002/jbmr.320 21254230 PMC3179345

[B13] Boyce B. F. Xing L. (2007). The rankl/rank/opg pathway. Curr. Osteoporos. Rep. 5, 98–104. 10.1007/s11914-007-0024-y 17925190

[B14] Boyle W. J. Simonet W. S. Lacey D. L. (2003). Osteoclast differentiation and activation. Nature 423, 337–342. 10.1038/nature01658 12748652

[B15] Brömme D. Lecaille F. (2009). Cathepsin K inhibitors for osteoporosis and potential off-target effects. Expert Opin. Investig. Drugs 18, 585–600. 10.1517/13543780902832661 19388876 PMC3110777

[B16] Calvaruso M. Militello C. Minafra L. La Regina V. Torrisi F. Pucci G. (2021). Biological and mechanical characterization of the random positioning machine (RPM) for microgravity simulations. Life 11, 1190. 10.3390/life11111190 34833068 PMC8619501

[B17] Carnovali M. Luzi L. Banfi G. Mariotti M. (2016). Chronic hyperglycemia affects bone metabolism in adult zebrafish scale model. Endocrine 54, 808–817. 10.1007/s12020-016-1106-3 27696252

[B18] Carnovali M. Banfi G. Mariotti M. (2021). Age-dependent modulation of bone metabolism in zebrafish scales as new model of Male osteoporosis in lower vertebrates. Geroscience 43, 927–940. 10.1007/s11357-020-00267-0 32997256 PMC8110640

[B19] Carvajal-Agudelo J. D. McNeil A. Franz-Odendaal T. A. (2023). Effects of simulated microgravity and vibration on osteoblast and osteoclast activity in cultured zebrafish scales. Life Sci. Space Res. (Amst) 38, 39–45. 10.1016/j.lssr.2023.05.002 37481306

[B20] Carvajal-Agudelo J. D. Eaton J. Franz-Odendaal T. A. (2024). Reduced ossification caused by 3D simulated microgravity exposure is short-term in larval zebrafish. Life Sci. Space Res. (Amst) 41, 127–135. 10.1016/j.lssr.2024.02.006 38670639

[B21] Chatziravdeli V. Katsaras G. N. Lambrou G. I. (2019). Gene expression in osteoblasts and osteoclasts under microgravity conditions: a systematic review. Curr. Genomics 20, 184–198. 10.2174/1389202920666190422142053 31929726 PMC6935951

[B22] Chen C. Jiang Y. Xu C. Liu X. Hu L. Xiang Y. (2016). Skeleton genetics: a comprehensive database for genes and mutations related to genetic skeletal disorders. Database 2016, baw127. 10.1093/database/baw127 27580923 PMC5006089

[B23] Chen Z. Song Z. Yang J. Huang J. Jiang H. (2019). Sp7/osterix positively regulates dlx2b and bglap to affect tooth development and bone mineralization in zebrafish larvae. J. Biosci. 44, 1–9. 31894108

[B24] Clark C. T. Smith K. K. (1993). Cranial osteogenesis in Monodelphis domestica (didelphidae) and macropus eugenii (macropodidae). J. Morphol. 215 (2), 119–149. 10.1002/jmor.1052150203 8445660

[B25] Corydon T. J. Schulz H. Richter P. Strauch S. M. Böhmer M. Ricciardi D. A. (2023). Current knowledge about the impact of microgravity on gene regulation. Cells 12, 1043. 10.3390/cells12071043 37048115 PMC10093652

[B26] Cubbage C. C. Mabee P. M. (1996). Development of the cranium and paired fins in the zebrafish *Danio rerio* (ostariophysi, cyprinidae). J. Morphol. 229, 121–160. 10.1002/(SICI)1097-4687(199608)229:2<121::AID-JMOR1>3.0.CO;2-4 29852585

[B27] Deng L. Lei D. Liu Y. Wang Q. Wen Z. Qiu J. (2017). Effects of simulated microgravity on vascular development in zebrafish. Mol. and Cell. Biomechanics 14, 171.

[B28] Dietrich K. Fiedler I. A. Kurzyukova A. López-Delgado A. C. McGowan L. M. Geurtzen K. (2020). Skeletal biology and disease modeling in zebrafish. J. Bone Mineral Res. 36, 436–458. 10.1002/jbmr.4256 33484578

[B29] Dougall W. C. Chaisson M. (2006). The RANK/RANKL/OPG triad in cancer-induced bone diseases. Cancer Metastasis Rev. 25, 541–549. 10.1007/s10555-006-9021-3 17180711

[B30] Drake M. T. Clarke B. L. Lewiecki E. M. (2015). The pathophysiology and treatment of osteoporosis. Clin. Ther. 37, 1837–1850. 10.1016/j.clinthera.2015.06.006 26163201

[B31] Eaton J. Carvajal-Agudelo J. D. Franz-Odendaal T. (2023). Comparison of effects of 2D and 3D simulated microgravity rotation on ossification in larval *Danio rerio* (zebrafish). Microgravity Sci. Technol. 35, 53. 10.1007/s12217-023-10077-6

[B32] Erlebacher A. Filvaroff E. H. Ye J.-Q. Derynck R. (1998). Osteoblastic responses to TGF-β during bone remodeling. Mol. Biol. Cell 9, 1903–1918. 10.1091/mbc.9.7.1903 9658179 PMC25433

[B33] Franke T. F. Hornik C. P. Segev L. Shostak G. A. Sugimoto C. (2003). PI3K/Akt and apoptosis: size matters. Oncogene 22, 8983–8998. 10.1038/sj.onc.1207115 14663477

[B34] Franz-Odendaal T. A. Edsall S. C. (2018). Long-term effects of simulated microgravity and vibration exposure on skeletal development in zebrafish. Stem Cells Dev. 27, 1278–1286. 10.1089/scd.2017.0266 29790426

[B35] Frost H. M. (1987). Bone “mass” and the “mechanostat”: a proposal. Anatomical Record 219 (1), 1–9. 10.1002/ar.1092190104 3688455

[B36] Fukumoto S. (2019). “FGF23 and bone and mineral metabolism,” in Bone regulators and osteoporosis therapy (Springer), 281–308. 10.1007/164_2019_330 31792685

[B37] Gillette-Ferguson I. Ferguson D. G. Poss K. D. Moorman S. J. (2003). Changes in gravitational force induce alterations in gene expression that can be monitored in the live, developing zebrafish heart. Adv. Space Res. 32, 1641–1646. 10.1016/S0273-1177(03)90405-4 15002421

[B38] Grimm D. Bauer J. Kossmehl P. Shakibaei M. Schöberger J. Pickenhahn H. (2002). Simulated microgravity alters differentiation and increases apoptosis in human follicular thyroid carcinoma cells. FASEB J. 16, 604–606. 10.1096/fj.01-0673fje 11919168

[B39] Han Y. Shao W. Zhong D. Ma C. Wei X. Ahmed A. (2021). Zebrafish mafbb mutants display osteoclast over-activation and bone deformity resembling osteolysis in MCTO patients. Biomolecules 11, 480. 10.3390/biom11030480 33806930 PMC8004647

[B40] Hang X. Li H. Ma W. Sun Y. (2011). “Effects of simulated-microgravity on development and p53 gene expression of zebrafish embryos,” in 2011 international conference on remote sensing, environment and transportation engineering (IEEE), 8273–8276.

[B41] He H. Wang C. Tang Q. Yang F. Xu Y. (2018). Possible mechanisms of prednisolone-induced osteoporosis in zebrafish larva. Biomed. and Pharmacother. 101, 981–987. 10.1016/j.biopha.2018.02.082 29635908

[B42] Ho N. C. Jia L. Driscoll C. C. Gutter E. M. Francomano C. A. (2000). A skeletal gene database. J. Bone Mineral Res. 15, 2095–2122. 10.1359/jbmr.2000.15.11.2095 11092392

[B43] Hofbauer L. C. Kuhne C. A. Viereck V. (2004). The OPG/RANKL/RANK system in metabolic bone diseases. J. Musculoskelet. Neuronal Interact. 4, 268–275. 15615494

[B44] Huang H. Wu C. K. Wu D. J. Liu W. H. Lee Y. S. Wu C. L. (2023). Apoptosis pathways and osteoporosis: an approach to genomic analysis. J. Gene Med. 25, e3555. 10.1002/jgm.3555 37461161

[B45] Kawai R. Sugisaki R. Miyamoto Y. Yano F. Sasa K. Minami E. (2023). Cathepsin K degrades osteoprotegerin to promote osteoclastogenesis *in vitro* . Vitro Cell. and Dev. Biology-Animal 59, 10–18. 10.1007/s11626-023-00747-5 36689044

[B46] Kim Y.-I. Lee S. Jung S. H. Kim H. T. Choi J. H. Lee M. S. (2013). Establishment of a bone-specific col10a1: GFP transgenic zebrafish. Mol. Cells 36, 145–150. 10.1007/s10059-013-0117-7 23852131 PMC3887955

[B47] Kim A. S. Girgis C. M. McDonald M. M. (2022). Osteoclast recycling and the rebound phenomenon following denosumab discontinuation. Curr. Osteoporos. Rep. 20, 505–515. 10.1007/s11914-022-00756-5 36201122 PMC9718877

[B48] Kitamura K. Takahira K. Inari M. Satoh Y. Hayakawa K. Tabuchi Y. (2013). Zebrafish scales respond differently to *in vitro* dynamic and static acceleration: analysis of interaction between osteoblasts and osteoclasts. Comp. Biochem. Physiol. A Mol. Integr. Physiol. 166, 74–80. 10.1016/j.cbpa.2013.04.023 23632157

[B49] Krishnan V. Bryant H. U. MacDougald O. A. (2006). Regulation of bone mass by wnt signaling. J. Clin. Invest 116, 1202–1209. 10.1172/JCI28551 16670761 PMC1451219

[B50] Lacey D. L. Boyle W. J. Simonet W. S. Kostenuik P. J. Dougall W. C. Sullivan J. K. (2012). Bench to bedside: elucidation of the OPG–RANK–RANKL pathway and the development of denosumab. Nat. Rev. Drug Discov. 11, 401–419. 10.1038/nrd3705 22543469

[B51] Lerebours A. Gonzalez P. Adam C. Camilleri V. Bourdineaud J. P. Garnier-Laplace J. (2009). Comparative analysis of gene expression in brain, liver, skeletal muscles, and gills of zebrafish (*Danio rerio*) exposed to environmentally relevant waterborne uranium concentrations. Environ. Toxicol. Chem. Int. J. 28, 1271–1278. 10.1897/08-357.1 19146232

[B52] Li B. Dewey C. N. (2011). RSEM: accurate transcript quantification from RNA-seq data with or without a reference genome. BMC Bioinforma. 12, 323. 10.1186/1471-2105-12-323 21816040 PMC3163565

[B53] Li N. Felber K. Elks P. Croucher P. Roehl H. H. (2009). Tracking gene expression during zebrafish osteoblast differentiation. Dev. Dynamics 238 (2), 459–466. 10.1002/dvdy.21838 19161246

[B54] Li L. Gu N. Dong H. Li B. Kenneth T. V. G. (2020). Analysis of the effects of acoustic levitation to simulate the microgravity environment on the development of early zebrafish embryos. RSC Adv. 10, 44593–44600. 10.1039/d0ra07344j 35517124 PMC9058438

[B55] Lien N. (2022). Differential gene expression analysis of zebrafish embryos exposed to simulated microgravity and insights into cellular effects.

[B56] Liu H. Ru N. Y. Cai Y. Lyu Q. Guo C. H. Zhou Y. (2022). The OPG/RANKL/RANK system modulates calcification of common carotid artery in simulated microgravity rats by regulating NF-κB pathway. Can. J. Physiol. Pharmacol. 100, 324–333. 10.1139/cjpp-2021-0329 34670103

[B57] Liu Z. Wang Q. Zhang J. Qi S. Duan Y. Li C. (2023). The mechanotransduction signaling pathways in the regulation of osteogenesis. Int. J. Mol. Sci. 24 (18), 14326. 10.3390/ijms241814326 37762629 PMC10532275

[B58] López Garzón N. A. Pinzón-Fernández M. V. Saavedra T J. S. Nati-Castillo H. A. Arias-Intriago M. Salazar-Santoliva C. (2025). Microgravity and cellular biology: insights into cellular responses and implications for human health. Int. J. Mol. Sci. 26, 3058. 10.3390/ijms26073058 40243850 PMC11988870

[B59] Lotinun S. Kiviranta R. Matsubara T. Alzate J. A. Neff L. Lüth A. (2013). Osteoclast-specific cathepsin K deletion stimulates S1P-dependent bone formation. J. Clin. Invest 123, 666–681. 10.1172/JCI64840 23321671 PMC3561821

[B60] Love M. Anders S. Huber W. (2014). Differential analysis of count data–the DESeq2 package. Genome Biol. 15, 10–1186.

[B61] Man J. Graham T. Squires-Donelly G. Laslett A. L. (2022). The effects of microgravity on bone structure and function. Npj Microgravity 8 (1), 9. 10.1038/s41526-022-00194-8 35383182 PMC8983659

[B62] Nagaraja M. P. Risin D. (2013). The current state of bone loss research: data from spaceflight and microgravity simulators. J. Cell Biochem. 114, 1001–1008. 10.1002/jcb.24454 23150462

[B63] Niu P. Zhong Z. Wang M. Huang G. Xu S. Hou Y. (2017). Zinc finger transcription factor Sp7/Osterix acts on bone formation and regulates col10a1a expression in zebrafish. Sci. Bull. (Beijing) 62, 174–184. 10.1016/j.scib.2017.01.009 36659402

[B64] Novack D. V. Teitelbaum S. L. (2008). The osteoclast: friend or foe? Annu. Rev. Pathol. Mech. Dis. 3, 457–484. 10.1146/annurev.pathmechdis.3.121806.151431 18039135

[B65] Pasqualetti S. Banfi G. Mariotti M. (2012). The zebrafish scale as model to study the bone mineralization process. J. Mol. Histol. 43, 589–595. 10.1007/s10735-012-9425-z 22661010

[B66] Pinton P. Giorgi C. Siviero R. Zecchini E. Rizzuto R. (2008). Calcium and apoptosis: ER-Mitochondria Ca2+ transfer in the control of apoptosis. Oncogene 27, 6407–6418. 10.1038/onc.2008.308 18955969 PMC2844952

[B67] Prasad B. Grimm D. Strauch S. M. Erzinger G. S. Corydon T. J. Lebert M. (2020). Influence of microgravity on apoptosis in cells, tissues, and other systems *in vivo* and *in vitro* . Int. Journal Molecular Sciences 21 (24), 9373. 10.3390/ijms21249373 33317046 PMC7764784

[B68] Quarles L. D. (2003). FGF23, PHEX, and MEPE regulation of phosphate homeostasis and skeletal mineralization. Am. J. Physiology-Endocrinology Metabolism 285, E1–E9. 10.1152/ajpendo.00016.2003 12791601

[B69] Riwaldt S. Corydon T. J. Pantalone D. Sahana J. Wise P. Wehland M. (2021). Role of apoptosis in wound healing and apoptosis alterations in microgravity. Front. Bioengineering Biotechnology 9, 679650. 10.3389/fbioe.2021.679650 34222218 PMC8248797

[B70] Robling A. G. Castillo A. B. Turner C. H. (2006). Biomechanical and molecular regulation of bone remodeling. Annu. Rev. Biomed. Eng. 8 (1), 455–498. 10.1146/annurev.bioeng.8.061505.095721 16834564

[B71] Rucci N. Rufo A. Alamanou M. Teti A. (2007). Modeled microgravity stimulates osteoclastogenesis and bone resorption by increasing osteoblast RANKL/OPG ratio. J. Cell Biochem. 100, 464–473. 10.1002/jcb.21059 16927271

[B72] Sasaki F. Hayashi M. Ono T. Nakashima T. (2021). The regulation of RANKL by mechanical force. J. Bone Mineral Metabolism 39, 34–44. 10.1007/s00774-020-01145-7 32889574

[B73] Sebastian A. Loots G. G. (2017). Transcriptional control of sost in bone. Bone 96, 76–84. 10.1016/j.bone.2016.10.009 27771382

[B74] Shao C. Chen H. Liu T. Pan C. (2025). The hippo pathway in bone and cartilage: implications for development and disease. Peer J. 13, e19334. 10.7717/peerj/19334 40292098 PMC12024444

[B75] Silva I. Branco J. C. (2011). Rank/Rankl/opg: literature review. Acta Reumatol. Port. 36, 209–218. 22113597

[B76] Takayanagi H. (2007). Osteoimmunology: shared mechanisms and crosstalk between the immune and bone systems. Nat. Rev. Immunol. 7, 292–304. 10.1038/nri2062 17380158

[B77] Theoleyre S. Wittrant Y. Tat S. K. Fortun Y. Redini F. Heymann D. (2004). The molecular triad OPG/RANK/RANKL: involvement in the orchestration of pathophysiological bone remodeling. Cytokine Growth Factor Rev. 15, 457–475. 10.1016/j.cytogfr.2004.06.004 15561602

[B78] Tyrina E. Yakubets D. Markina E. Buravkova L. (2024). Hippo signaling pathway involvement in osteopotential regulation of murine bone marrow cells under simulated microgravity. Cells 13, 1921. 10.3390/cells13221921 39594669 PMC11592674

[B79] Udagawa N. Koide M. Nakamura M. Nakamichi Y. Yamashita T. Uehara S. (2021). Osteoclast differentiation by RANKL and OPG signaling pathways. J. Bone Min. Metab. 39, 19–26. 10.1007/s00774-020-01162-6 33079279

[B80] Uva B. M. Masini M. A. Sturla M. Bruzzone F. Giuliani M. Tagliafierro G. (2002). Microgravity-induced apoptosis in cultured glial cells. Eur. J. Histochem. 46, 209–214. 10.4081/1681 12472115

[B81] Wang Y. Li Y. Chen Q. Liu Z. (2019). Long-term exposure of xenoestrogens with environmental relevant concentrations disrupted spermatogenesis of zebrafish through altering sex hormone balance, stimulating germ cell proliferation, meiosis and enhancing apoptosis. Environ. Pollut. 244, 486–494. 10.1016/j.envpol.2018.10.079 30366296

[B82] Wang Y. Xie Z. Kutschera E. Adams J. I. Kadash-Edmondson K. E. Xing Y. (2024). rMATS-turbo: an efficient and flexible computational tool for alternative splicing analysis of large-scale RNA-Seq data. Nat. Protoc. 19, 1083–1104. 10.1038/s41596-023-00944-2 38396040

[B83] Wen G. (2017). “A simple process of RNA-Sequence analyses by Hisat2, htseq and DESeq2,” in Proceedings of the 2017 international conference on biomedical engineering and bioinformatics, 11–15.

[B84] Wiren K. M. Toombs A. R. Semirale A. A. Zhang X. (2006). Osteoblast and osteocyte apoptosis associated with androgen action in bone: requirement of increased Bax/Bcl-2 ratio. Bone 38, 637–651. 10.1016/j.bone.2005.10.029 16413235

[B85] Xu D. Lv J. He L. Fu L. Hu R. Cao Y. (2018). Scribble influences cyst formation in autosomal‐dominant polycystic kidney disease by regulating hippo signaling pathway. FASEB J. 32, 4394–4407. 10.1096/fj.201701376RR 29529391

[B86] Yang X. Sun L.-W. Liang M. Wang X.-N. Fan Y.-B. (2015). The response of wnt/ß-catenin signaling pathway in osteocytes under simulated microgravity. Microgravity Sci. Technol. 27, 473–483. 10.1007/s12217-015-9439-8

[B87] Yue J. López J. M. (2020). Understanding MAPK signaling pathways in apoptosis. Int. J. Mol. Sci. 21. 10.3390/ijms21072346 32231094 PMC7177758

