# Supplementary material for: Transcriptomic profiling and RANKL/RANK/OPG-mediated osteoclastogenesis in zebrafish larvae under simulated microgravity conditions
Source: Front Cell Dev Biol. 2026 May 4;14:1786373. doi: 10.3389/fcell.2026.1786373 (PMC13181342; doi:10.3389/fcell.2026.1786373)
Supplement: Supplementary file 1 [file DataSheet3.docx]

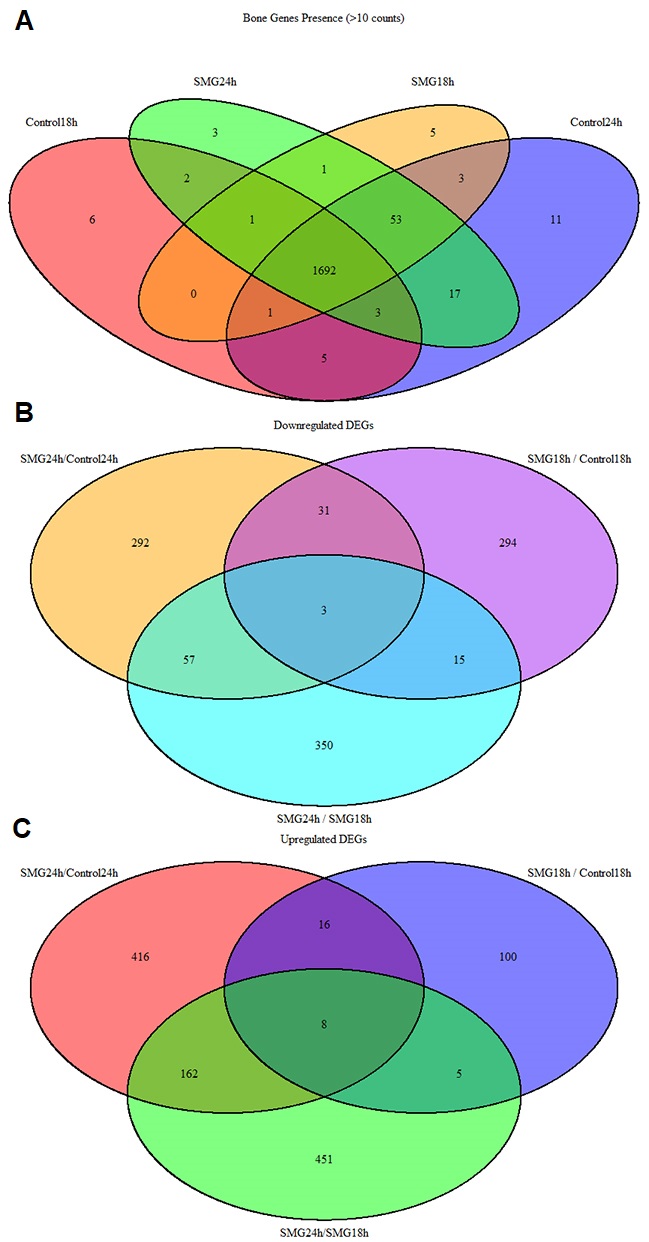


**Supplementary Data 3 - Overlap of differentially expressed bone-related genes across experimental conditions.**

Figure 7. Venn diagrams showing the overlap of differentially expressed bone-related genes across experimental conditions. (A) Bone-related gene presence: Genes from the curated bone-related gene list detected above the threshold (>10 average read counts) in Control18h, Control24h, SMG24h, and SMG18h. The diagram highlights both unique and shared genes across conditions. (B) Downregulated genes: Genes significantly downregulated in the same comparisons. The diagram illustrates unique and shared downregulated genes among the conditions. (C)Upregulated genes: Genes significantly upregulated in SMG24h vs Control24h, SMG18h vs Control18h, and SMG24h vs SMG18h. The diagram shows both unique and shared genes among the three comparisons. The center indicates genes upregulated in all conditions.
